# A modular cementless stem vs. cemented long-stem prostheses in revision surgery of the hip: A population-based study from the Swedish Hip Arthroplasty Register

**DOI:** 10.3109/17453674.2011.566145

**Published:** 2011-04-05

**Authors:** Rüdiger J Weiss, André Stark, Johan Kärrholm

**Affiliations:** ^1^Department of Molecular Medicine and Surgery, Section of Orthopaedics and Sports Medicine, Karolinska Institutet, Karolinska University Hospital, Stockholm; ^2^Department of Clinical Sciences, Karolinska Institutet, Danderyd Hospital, Stockholm; ^3^Department of Orthopaedics, Division of Clinical Sciences at Sahlgrenska Academy, Gothenburg University, Gothenburg, Sweden

## Abstract

**Background and purpose:**

Modular cementless revision prostheses are being used with increasing frequency. In this paper, we review risk factors for the outcome of the Link MP stem and report implant survival compared to conventional cemented long-stem hip revision arthroplasties.

**Patients and methods:**

We used data recorded in the Swedish Hip Arthroplasty Register. 812 consecutive revisions with the MP stem (mean follow-up time 3.4 years) and a control group with 1,073 cemented long stems (mean follow-up time 4.2 years) were included. Kaplan-Meier analysis was used to determine implant survival. The Cox regression model was used to study risk factors for reoperation and revision.

**Results:**

The mean age at revision surgery for the MP stem was 72 (SD 11) years. Decreasing age (HR = 1.1, 95% CI: 1–1.1), multiple previous revisions (HR = 2.6, 95% CI: 1.1–6.2), short stem length (HR = 2.4, 95% CI: 1.1–5.2), standard neck offset (HR = 5, 95% CI: 1.5–17) and short head-neck length (HR = 5.3, 95% CI 1.4–21) were risk factors for reoperation. There was an overall increased risk of reoperation (HR = 1.7, 95% CI: 1.3–2.4) and revision (HR = 1.9, 95% CI: 1.2–3.1) for the MP prostheses compared to the controls.

**Interpretation:**

The cumulative survival with both reoperation and revision as the endpoint was better for the cemented stems with up to 3 years of follow-up. Thereafter, the survival curves converged, mainly because of increasing incidence of revision due to loosening in the cemented group. We recommend the use of cemented long stems in patients with limited bone loss and in older patients.

During the past decade, cementless fixation has been increasingly used in revision hip arthroplasty both on the acetabular and the femoral side (Swedish Hip Arthroplasty Register). Several designs of tapered and modular fluted stems have been developed. The aim is to provide immediate axial and rotational stability distally in the femur, where the bone is less compromised by the loosening process (Wirtz et al. 2000, Kwong et al. 2003, Schuh et al. 2004, McInnis et al. 2006, Tamvakopoulos et al. 2007, Rodriguez et al. 2009, Weiss et al. 2009). In revisions, bone loss and deformity are not always predictable. Modular, distally fixed stems might facilitate the use of different strategies to reconstruct the femur and they are also an alternative in less complex cases, which might explain their increasing popularity.

We studied one of these designs, the MP hip reconstruction prosthesis (Waldemar Link, Germany) on a nationwide basis in Sweden. This design was chosen because it has been the most frequently used one and has had the longest follow-up. By studying reoperations and revisions, we wanted to identify risk factors for the outcome of the MP stem using any further operation of the same hip after the index procedure (reoperation), or exchange of parts (or the entire prosthesis), or implant removal (revision) as outcome parameters. Patients listed in the hip register who were revised with a long cemented stem during the same period were studied for comparison.

## Patients and methods

### Source of data

The Swedish Hip Arthroplasty Register collects individual-based information for hip replacement surgery on a nationwide basis in Sweden (Malchau et al. 2002). Data on all primary and revision total hip replacement operations are collected and are identifiable by the unique social security numbers of the patients. Demographic data and details of indications for reoperation or revision, surgical technique, and the type of prosthetic components inserted are recorded. The register covers about 98% of all primary hip replacement surgical procedures in Sweden, whereas the coverage of revision hip arthroplasties has been estimated to be 94% (Soderman et al. 2000).

**Figure 1.**
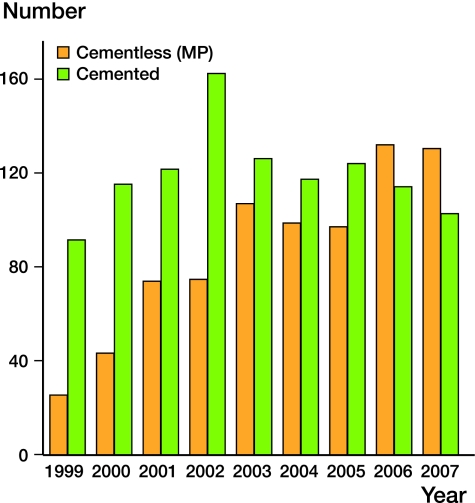
Number of operations with the cementless MP hip revision stem and cemented long-stem prostheses in Sweden during 1999 and 2007.

The MP stem was chosen because this design has been the most commonly used modular revision stem in Sweden (72% of all recorded cases). The first MP stem used as revision prosthesis was inserted in 1994, but until 1999 this stem was used in small numbers (< 10 annually). We studied operations performed until 2007. To obtain an approximately equal follow-up in the study population as in the control population, patients were recruited from 1999 (Figure 1). Only stems corresponding to the 3 most commonly used designs (Lubinus, Exeter, and Spectron) and longer than the corresponding largest standard stem were included. In patients with bilateral revisions, both sides were included in the analysis, as other studies have shown that this does not influence the risk of revision (Lie et al. 2004, Hailer et al. 2010).

### Implants

**Figure 2.**
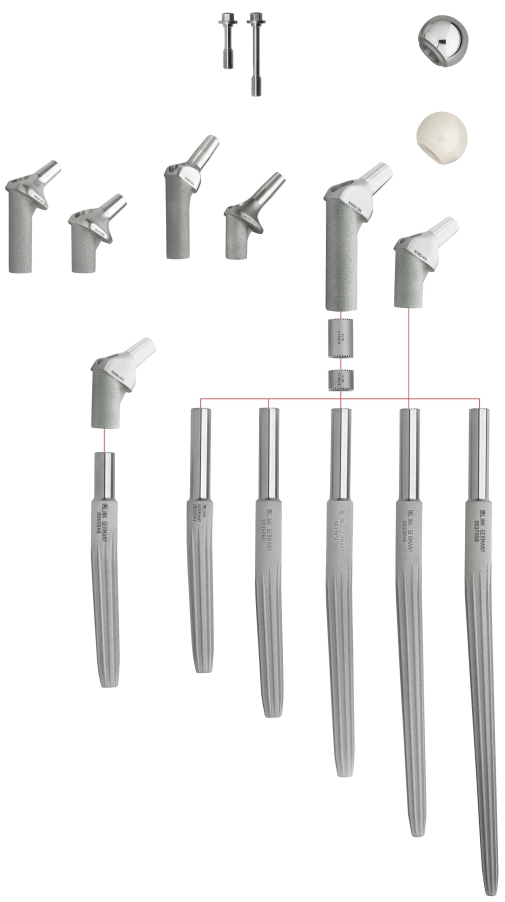
The Link MP system.

*Cementless MP revision arthroplasty.* The modular MP stem is made of titanium alloy and has a microporous surface (Figure 2). The tapered distal part has a fluted geometry with a 3° angular bow to accommodate the femoral curvature. After reaming, the implant is impacted into the femur until rigid stability to axial and torsional testing is achieved. Varying stem lengths and diameters allow independent fitting to the diaphyseal part of the femur. The proximal part is available in various sizes and shapes, and can be used with 2 offsets and caput (femoral head) – collum (neck) – diaphysis (CCD) angles (126° and 135°). Depending on size, the distal part contains 8 longitudinal flutes (stem size 12–16) or 10 longitudinal flutes (stem size 18–25) to support rotational stability and to reduce the stiffness of the implant. The length of the assembled implant can be adjusted by the use of spacers (Link 2005).

*Cemented long-stem arthroplasties (control group).* The control group included femoral components such as Lubinus (length 170–350 mm; Waldemar Link, Hamburg, Germany), the Spectron revision hip system (165–225 mm; Smith & Nephew Inc., Memphis, TN) and the Exeter long stem (200–300 mm; Stryker, Mahwah, NJ). Patients operated with impaction grafting or who—according to the records in the register—had received any other type of bone graft were excluded.

### Patients

*MP stem.* 787 patients (429 males, 55%) had been revised with a Link MP hip stem during the study period. 25 patients received bilateral MP prostheses, resulting in a total of 812 operated hips. The mean age at revision surgery was 72 (SD 11, range 26–96) years. 46% were older than 75 years at the index operation (Table 1). The mean follow-up time was 3.4 (SD 2.9, range 0–13) years. At the index operation, most hips were revised due to aseptic loosening (57%) or periprosthetic fractures (26%). The primary diagnosis of this patient cohort was dominated by primary osteoarthritis (69%) followed by inflammatory arthritis (10%) (Table 1).

**Table 1.** Demographics

	MP stem	Cemented long stem
Total number of cases	812	1,073
Male	443 (55%)	544 (51%)
Female	369 (45%)	529 (49%)
Age at index operation		
< 65 years	190 (23%)	114 (11%)
65–75 years	248 (31%)	288 (27%)
> 75 years	374 (46%)	671 (62%)
Mean (SD) years	72 (11)	76 (9)
Age at primary total joint arthroplasty		
Mean (SD) years	59 (12)	64 (10)
Diagnosis at primary total joint arthroplasty		
Primary osteoarthritis	557 (69%)	798 (74%)
Inflammatory arthritis	82 (10%)	86 (8%)
Fracture	72 (9%)	118 (11%)
Childhood disease	51 (6%)	30 (3%)
Other	50 (6%)	41 (4%)
Diagnosis at index operation		
Aseptic loosening	463 (57%)	676 (63%)
Periprosthetic fracture	208 (26%)	230 (21%)
Deep infection	3 (<1%)	16 (1%)
Dislocation	11 (1%)	16 (2%)
Other	127 (16%)	135 (13%)
Surgical procedures before the index operation		
1	413 (51%)	678 (63%)
2	225 (28%)	237 (22%)
3	100 (12%)	103 (10%)
≥ 4	74 (9%)	55 (5%)
Component exchange before index operation		
0	571 (70%)	875 (82%)
1	190 (24%)	156 (14%)
2	42 (5%)	32 (3%)
> 2	9 (1%)	10 (1%)

The overall number of revisions with the MP stem increased during the study period (Figure 1). In half of the cases (51%), the revision with the MP stem was the first reoperation after implantation of the primary arthroplasty. 30% had undergone 1 or more previous revisions (Table 1).

Most commonly, stem diameters of 16 mm (29%) or 18 mm (28%) and stems with a length of 250 mm (43%) or 210 mm (29%) were used (Table 2). During the study period, there was a trend to use thicker stems (data not included in the analyses).

**Table 2.** MP stems and cemented long stems

MP stem	n (%)
Total	812
Stem diameter	
12 mm	9 (1)
14 mm	71 (9)
16 mm	239 (29)
18 mm	231 (28)
20 mm	154 (19)
22.5 mm	44 (5)
25 mm	9 (1)
Custom made	1 (0.1)
Missing	54 (7)
Stem length	
160 mm	3 (0.4)
180 mm	67 (8)
210 mm	234 (29)
250 mm	349 (43)
290 mm	89 (11)
330 mm	15 (2)
Custom-made	1 (0.1)
Missing	54 (7)
Femoral head size	
22 mm	79 (10)
28 mm	607 (75)
32 mm	69 (8)
Other	2 (0)
Missing	55 (7)
Cemented long stem	
Total	1,073
Lubinus SP II (≥ 170 mm)	610 (57)
Exeter long-stem (≥ 200 mm)	248 (23)
Spectron revision hip system (≥ 165 mm)	215 (20)
Femoral head size	
22 mm	62 (6)
28 mm	622 (58)
32 mm	66 (6)
Other	1 (0)
Missing	322 (30)

*Cemented long stems (control group).* During the selected time period, there were 1,056 patients (534 males, 51%) operated with cemented long-stem prostheses in the register (1,073 hips). Their mean age at the index operation was 76 (SD 9, range 27–101) years (Table 1). 62% of the patients were older than 75 years at the index operation. The mean follow-up time was 4.2 (SD 2.5, range 0–9) years. The reasons for revision were aseptic loosening and periprosthetic fracture in 63% and 21% of the cases (Table 1). The Lubinus SP II was most commonly used (57%), followed by the Exeter long stem (23%) and the Spectron revision hip system (20%, Table 2).

### Statistics

Mean values and standard deviations (SDs) were used as descriptive statistics. The endpoint for survival was defined either as reoperation or revision. The term reoperation included all types of new surgical procedures in the same hip following the index operation. Revision was defined as one type of reoperation during which one, several, or all parts of the prosthesis were exchanged or extracted.

The Cox multiple-regression model was used to study risk factors for reoperation and revision related to the patient, to the implant, and to the surgical technique. The results were expressed as hazard ratios (HRs) with corresponding 95% confidence intervals (CIs). The factors studied in the Cox model were: age at primary and revision surgery, sex, diagnosis at the first surgical procedure and at the index operation, and number of surgical procedures before the index operation. In a second analysis including 755 MP stem operations with complete data, any association between implant component characteristics (head size, stem length and width, use of extra offset, neck length and combined offset (neck length + offset)), and the risk of further reoperation/revision was studied. The assumption of proportional hazards was investigated by hazard function plots and log-log plots of all covariates. No signs of insufficient proportionality were detected. All log-log plots ran strictly parallel for all covariates.

Kaplan-Meier analysis was used to construct the cumulative survival for both reoperation and revision as the criterion of failure. The Cox multiple-regression model was used to study differences between groups and to adjust for potential confounding factors. The level of significance was set at p ≤ 0.05. All statistical analyses were performed using the PASW statistics package version 18 (SPSS Inc., Chicago, IL).

## Results

### Reoperations and revisions of the MP prosthesis

The overall failure rate leading to a reoperation was 11% (93/812). The overall revision rate of the MP prosthesis, including both the proximal and the distal part of the implant, was 5% (39/812). Revisions were mainly due to dislocation (n = 17), followed by aseptic loosening (n = 6) and deep infection (n = 5) (Table 3). In 23 hips, only the proximal part was exchanged or adjusted. In 16, the entire stem was exchanged or extracted. In 18 cases, only the cup or liner was exchanged and in 8 cases only the femoral head was exchanged.

**Table 3.** Reasons for revision

	MP stem	Cemented long stem
Dislocation	17	2
Aseptic loosening	6	19
Deep infection	5	4
Periprosthetic fracture	2	7
Technical reasons	3	–
Implant fracture	1	–
Other	5	–

Cox regression analysis revealed that decreasing age (HR = 1.1, CI: 1–1.1), multiple previous revisions (HR = 2.6, CI: 1.1–6.2), a short stem length (HR = 2.4, CI: 1.1–5.2), a standard neck offset (HR = 5, CI: 1.5–17), and a short head-neck length (HR = 5.3, CI: 1.4–21) were independent risk factors for reoperation (Table 4).

**Table 4.** Risk of reoperation and revision of the MP stem (Cox regression analysis)

Factor	HR (95% CI)	p-value
Reoperation		
Decreasing age at primary hip arthroplasty	1.1 (1.0–1.1)	0.02
Number of revisions prior to the index operation (≥ 2 versus 1)	2.6 (1.1–6.2)	0.04
Short stem length (160–180 versus 250 mm)	2.4 (1.1–5.2)	0.03
Standard versus high neck offset (XXL)	5.0 (1.5–17)	0.009
Short (49–51.5 mm) versus long head-neck length (58–60 mm)	5.3 (1.4–21)	0.02
Revision		
Decreasing age at primary hip arthroplasty	1.1 (1.0–1.2)	0.03
Number of revisions prior to the index operation (≥ 2 versus 1)	3.8 (1.0–15)	0.05
Short stem length (160–180 versus 250 mm)	4.1 (1.4–12)	0.01

HR: hazard ratio. HRs are adjusted for age at primary and revision surgery, sex, diagnosis at primary and revision surgery, number of prior revisions, and different implant components

Risk factors for the exchange of several or all parts of the prosthesis (revision) were decreasing age (HR = 1.1, CI: 1–1.2), multiple previous revisions (HR = 3.8, CI: 1–14.7), and a short-stem length (HR = 4.1, CI = 1.4–12).

### Survival

Cox regression analysis revealed an increased risk of reoperation (adjusted HR = 1.7; CI: 1.3–2.4; p = 0.001) and revision (adjusted HR = 1.9, CI: 1.2–3.1; p = 0.008) for the cementless MP prosthesis compared to the cemented long stems.

**Figure 3.**
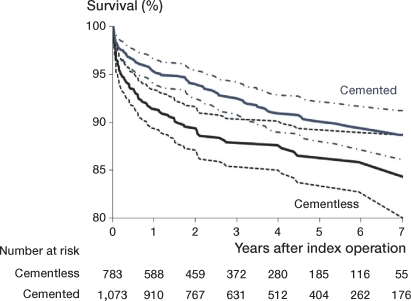
Kaplan-Meier analysis (with 95% confidence intervals) of the Link MP hip stem and cemented revision long-stem prostheses with reoperation as the endpoint.

**Figure 4.**
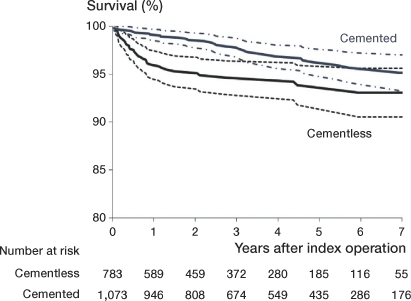
Kaplan-Meier analysis (with 95% confidence intervals) of the Link MP hip stem and cemented revision long-stem prostheses with revision as the endpoint.

The cumulative survival with both reoperation and revision as the endpoint was better for the cemented long-stem prostheses with up to 3 years follow-up. There was no difference between the MP group and the cemented group at 3 years and later (Figures 3 and 4).

**Figure 5.**
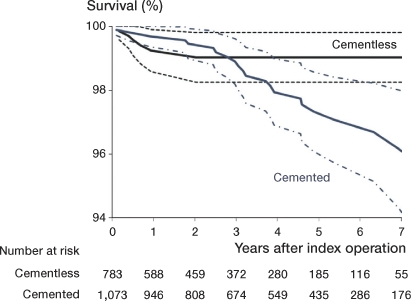
Kaplan-Meier-analysis (with 95% confidence intervals) of the Link MP hip stem and cemented revision long-stem prostheses with aseptic loosening as the endpoint.

During the entire observation time, cemented stems were mainly revised because of loosening (19 of 32 revisions of long cemented stems), whereas this reason was less common for revision of the MP stem (6 of 39 revisions). In the MP group, revision because of loosening occurred early, whereas revision of cemented long stems due to loosening was more evenly distributed in time (Figure 5).

## Discussion

We found a crude revision rate of the MP prosthesis of 5% after a mean follow-up of 3.4 years. More than half of these were adjustments or exchange of the proximal part of the modular stem. The risk of revision decreased with increasing stem length. Compared to a long cemented stem, we did not find any beneficial effects regarding either reoperation or revision (at least not in the short term). In the Cox regression model, bias caused by demographic factors, reasons for revision, and number of previous operations could be partly compensated for. Other confounders such as degree of bone deficiency and the general condition and activity of the patient were unknown.

There are several important goals in revision total hip arthroplasty such as implant stability, restoration of bone stock, and preservation of leg length. Fluted, tapered, grit-blasted titanium stems have shown mostly favorable results in the last 2 decades. Most newer stem designs are based on the cementless Wagner monoblock prosthesis (Bohm and Bischel 2001). However, modular implants such as the MP system have several advantages over monoblock prostheses. The distal and still intact part of the femur can be used for fixation. In our cementless cohort, all revisions due to loosening occurred during the first 2 years after the index operation, whereas in the cemented group 13 of 17 hips were revised after 2 years. The early failure rate of the MP stems due to loosening could reflect poor bone stock in these cases, but could also be due to poor surgical technique and the use of stems that are too thin and short, or a combination of these factors.

Several studies have found comparable early revision rates for the MP or similar stem designs. Survival rates of between 94% and 97% after a mean follow-up time of 2 to 4.2 years have been reported (Kwong et al. 2003, Schuh et al. 2004, Sporer and Paprosky 2004, McInnis et al. 2006, Ovesen et al. 2010, Rodriguez et al. 2009). Rodriguez et al. (2009) followed 102 hips for a mean of 3.3 years. The authors found a revision rate of 5% (n = 4) due to stem migration, implant fracture, and periprosthetic fracture. Kwong et al. (2003) noted a revision rate of 3% in 143 patients after a mean follow-up of 3.3 years. Sporer and Paprosky (2004) followed 16 patients with Paprosky type III and IV femoral defects that had been operated with a modular revision prosthesis, for an average of 2 years. 1 patient required femoral re-revision during this time period. McInnis et al. (2006) reported a 4% revision rate and a reoperation rate of 11% in 70 patients after an average of 3.9 years. Ovesen et al. (2010) noted a stem revision rate of 3% in 125 cases after an average follow-up time of 4.2 years.

We have previously reported on clinical and radiographic results of 90 cases that were revised with the MP stem with a minimum follow-up of 5 years. Even though the results were satisfying concerning pain reduction, survival, and stem migration, the dislocation rate was high. The only risk factor for dislocation that could be identified was a small prosthesis head (22 mm) (Weiss et al. 2011). In the present series, 10% of the cases in the MP group and 6% of the controls had a prosthesis with a head size of 22 mm. Dislocation was the most common reason for revision in the present series. The modularity of a stem means that the anteversion, offset, or stem length can easily be adjusted in cases with repeated dislocations. In the cemented group, only 2 of 32 cases were revised for the same reason. It may be that these cases were more commonly treated with closed reduction or with cup revision. We could not explore this issue further because closed reductions are not recorded in the register.

Restoration of the mechanics of the hip by balancing soft tissues and ensuring adequate femoral offset is essential. As expected, multiple prior surgical procedures—which may result in bone and soft-tissue deficiency—were an independent risk factor for further surgery. Previous hip surgery increases the risk of instability, leading to higher dislocation rates. Correct choice of implant components is essential for a good clinical outcome. Inadequate offset may result in failure to balance the soft tissues and alter the abductor muscle tension, with subsequent instability and increased risk of dislocation. In our series, the types of proximal implant components were not factors giving an increased revision rate in the Cox model. However, a shorter stem length increased the risk of further surgery. This may be explained by insufficient distal stem-bone anchorage of the prosthesis.

Several factors may contribute to better survival of cemented revision prostheses compared to the modular MP stem, as seen in our analysis. An earlier report from the register showed better survival of cemented revision stems compared to the cementless Wagner stem during 1990 and 2000. Moreover, there was better improvement in survival for the cemented stems during the study period (Malchau et al. 2002). The choice between cemented and cementless fixation involves many considerations, e.g. the anatomy of the femur, the degree of bone destruction, the age and general health of the patient, and surgeon's preference. In many countries, cementless fixation is the preferred choice for femoral revision components. In Sweden, there is an increasing trend to use more cementless total hip arthroplasties, both primarily and for revision. Still, in 2008 most of the implants used were cemented (Swedish Hip Arthroplasty Register).

By tradition, many Swedish surgeons are more familiar with the cemented technique. Optimum selection of implant sizes and the insertion of a modular cementless revision prosthesis has a learning curve. In the transition from a cemented revision implant to a fluted tapered stem, there is the risk of using sizes that are too small. This problem may be more common in low-volume hospitals. The dislocation problem with the modular stem may not only be related to its design. As an effect of poor bone stock or insufficient surgical technique, some stems may show an early period of subsidence and retroversion, thereafter achieving sufficient fixation. This early subsidence and change of stem position may, however, cause some instability of the joint, resulting in dislocation.

The results of cemented femoral revisions were rather poor in the 1980s (Kavanagh et al. 1985, Pellicci et al. 1985). However, more recent results with the use of second- and third-generation cementing techniques have shown an improved clinical outcome (Stromberg and Herberts 1994, Mulroy and Harris 1996, Hultmark et al. 2000, Lieberman 2005). These results and our own findings show that cemented femoral revision can be successful, at least in the short term. It should be noted that by definition, the control group received stems longer than the corresponding standard. When re-cementing a revision stem, it is important that the stem reaches at least one cortical width distal to the previous implant (Retpen and Jensen 1993, Hultmark et al. 2000). Even though we did not perform a radiographic analysis, our selection criteria probably favored the selection of such cases.

We chose the Lubinus, Exeter, and Spectron cemented long-stem prostheses as a control group, as they comprise most of the cemented stems used in revision hip arthroplasty in Sweden (Swedish Hip Arthroplasty Register). We did not include cemented revisions with impaction bone grafting, as the Link MP stem is implanted without the use of this technique. The information in the Swedish Register about the use of impaction grafting with dedicated instruments is not always complete, and in a substantial number of cases it would leave uncertainties about the way the bone graft was actually used.

Our results may also be seen in terms of increasing healthcare costs. We performed a rough estimation of implant costs and compared a standard MP prosthesis with a standard cemented long-stem arthroplasty (Lubinus) including bone cement, bone cement mixing system, and cement restrictor. From this, we estimated that the expenses associated with the MP prosthesis are approximately 70% higher.

Potential weaknesses of our study include the short follow-up. However, our aim was to describe the overall use on a nationwide basis rather than to perform a long-term clinical follow-up, and to identify potential risk factors for early failures. An obvious flaw of registry-derived data is the lack of information on clinical outcome and radiographic information. At present, the Swedish Hip Arthroplasty Register does not contain data regarding bone defects, which may have been different in the MP and control cohort. Cementless implants with fixation distally in the femoral canal have been successfully used in cases with moderate-to-severe bone loss. Differences in patient selection could therefore at least partly explain the fact that we found better early survival for cemented revision hip arthroplasties.

In conclusion, the MP modular tapered stem showed reduced early survival compared to recementing of a long-stem prosthesis. We therefore recommend the use of cemented long stems in patients with limited bone loss and in older patients.
